# Increased Population of CD40+ Fibroblasts Is Associated with Impaired Wound Healing and Chronic Inflammation in Diabetic Foot Ulcers

**DOI:** 10.3390/jcm11216335

**Published:** 2022-10-27

**Authors:** Joshua Patrick Bungalon Littig, Rebecca Moellmer, Adrienne M. Estes, Devendra K. Agrawal, Vikrant Rai

**Affiliations:** 1 College of Podiatry, Western University of Health Sciences, Pomona, CA 91766, USA; 2 Department of Translational Research, Western University of Health Sciences, Pomona, CA 91766, USA

**Keywords:** diabetic foot ulcers, nonhealing, chronic inflammation, immune cells, fibroblasts, phenotypic switch

## Abstract

Despite the advancement in the treatment, nonhealing diabetic foot ulcers (DFUs) are an important clinical issue accounting for increased morbidity and risk of amputation. Persistent inflammation, decreased granulation tissue formation, decreased neo-angiogenesis, and infections are common underlying causes of the nonhealing pattern. Fibroblasts play a critical role in granulation tissue formation and angiogenesis and mediate wound healing how fibroblasts regulate inflammation in nonhealing DFUs is a question to ponder. This study aims to investigate the expression of a de-differentiated subpopulation of fibroblasts which are CD40+ (secretory fibroblasts) and increased secretion of IL-6 and IL-8 but have never been reported in DFUs. We characterized 11 DFU tissues and nearby clean tissues histologically and for the presence of inflammation and CD40+ fibroblasts using immunohistochemistry and RT-PCR. The results revealed significantly increased density of CD40+ fibroblasts and differential expression of mediators of inflammation in DFU tissues compared to clean tissue. Increased expression of IL-6, IL-1β, and TNF-α in DFU tissues along with CD40+ fibroblast suggest that CD40+ fibroblasts in DFUs contribute to the chronicity of inflammation and targeting fibroblasts phenotypic switch to decrease secretory fibroblasts may have therapeutic significance to promote healing.

## 1. Introduction

Type 2 diabetes mellitus, T2DM, is a metabolic syndrome characterized by chronic hyperglycemia due to defects in the activity and insulin release. T2DM increases the risk of developing multiple burdensome complications for the patients and expenditures toward the healthcare system. Major complications for T2DM include atherosclerosis, retinopathy, nephropathy, and neuropathy. Neuropathic loss of sensation in the feet combined with external trauma or deformities can result in nonhealing ulcers, which increases the risk of lower limb amputations 15-fold compared to non-diabetic populations [1].

The physiological healing process is altered by the prolonged release of pro-inflammatory cytokines, including IL-6, TNF-α, IL-1, and IL-8, and the inappropriately persistent recruitment of immune cells. These factors hold the wound in the inflammatory phase without progressing to the resolution phase [2,3,4,5]. Studies have demonstrated the upregulation of IL-8 in diabetic wounds compared to non-diabetic populations [5]. In physiologic wound healing, inflammatory cytokines attract immune cells such as neutrophils and macrophages to the wound site for bacteria clearance, apoptosis of necrotic tissue, and disposal of cellular debris. This will lead to wound healing. However, prolonged expression of proinflammatory cytokines leads to the continuous infiltration of immune cells and chronic inflammation [6,7]. Despite the advancement in diabetic foot ulcer (DFU) treatment and care with wound debridement, off-loading, using antibiotics, and bandaging, there is a risk of lower limb amputation with relative risk between diabetic and non-diabetic patients between 7.4 and 41.3 and the worldwide prevalence of diabetes mellitus of 8.8% [8]. Over half of the major leg amputations performed every year in the United States are attributable to diabetes mellitus and peripheral artery disease [9,10,11]. Thus, an utmost need to develop better treatment strategies for nonhealing DFUs is vital because the underlying molecular mechanism in DFUs may vary from chronic ulcers due to persistent hyperglycemia in diabetes.

Fibroblasts play a crucial role in wound healing, and multiple studies have reported that dysregulated function and proliferation of fibroblasts contribute to a nonhealing pattern of wound healing [2,12]. Following the inflammatory phase is the proliferative phase, wherein fibroblasts activate and migrate to the injury site to form granulation tissue. The fibroblasts differentiate and take on an α-smooth muscle actin (α-SMA) phenotype, becoming myofibroblasts that facilitate the formation of extracellular matrix (ECM) remodeling, providing contractile properties to the wound site and the maturation of granulation tissue. As granulation tissue matures, distinct blood vessels form, capillary density falls, collagen content changes, and myofibroblasts become sparser at the site [12,13]. As tissue remodeling resolves, macrophages return to a passive state, and myofibroblasts undergo apoptosis [14]. The normal physiologic healing process is disrupted in diabetes-associated chronic inflammation. Elevated release of Toll-like receptor 9 (TLR9) induced pro-inflammatory molecules such as S100A8 and IL-8 from fibroblasts and immune cells continue to promote immune cell recruitment, activation, and inflammation [7,13]. 

These results suggest that fibroblasts’ plasticity and heterogeneity play a critical role in the nonhealing pattern of DFUs and that IL-8 secretion is associated with it. Moreover, increased secretion of IL-8 and IL-6 by fibroblasts on IL-1 and TNF-α stimulation suggests that fibroblasts secrete these cytokines in an inflammatory environment and the possibility of changing fibroblast phenotype to a secretory (CD40+) subtype [15,16]. Since these cytokines and chronic inflammation play a critical role in the nonhealing of DFUs, it is imperative to explore the presence of the secretory phenotype of fibroblasts in nonhealing DFUs and the change of fibroblast phenotype in presence of hyperglycemia towards secretory phenotype. Five different types of fibroblast populations in nonhealing wounds have been reported [5]; however, the presence of CD40+ fibroblast population in DFU samples and the possible phenotype switch to secretory phenotype have not been reported. In this study, we aimed to characterize the tissues from DFUs from human patients for inflammation and CD40+ subpopulation of fibroblasts phenotype (secretory fibroblasts). This study hypothesized that an increased population of CD40+ fibroblasts contribute to chronic inflammation and nonhealing and fibroblasts switching to an increased CD40+ fibroblasts population will attenuate the myofibroblasts population proportionately. Thus, targeting chronic inflammation and fibroblast phenotype switch will be of therapeutic significance.

## 2. Materials and Methods

**Figure 1:**
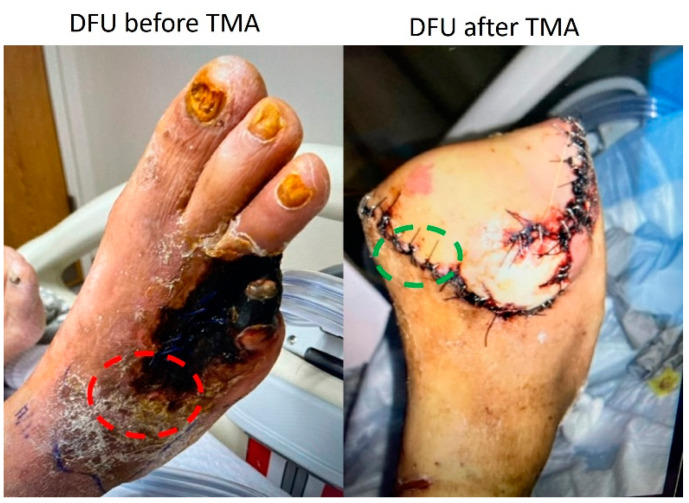
Tissues from the diabetic foot ulcer area (DFU, red circle) and clean samples near the margin of the wound (green circle) were collected from the human patient undergoing transmetatarsal amputation (TMA).

Patient selection and tissue collection: All patients undergoing transmetatarsal amputation (TMA) due to DFU with osteomyelitis were enrolled for the ongoing study with IRB approval number 1787616-2 by the Institutional Board of Riverside University Hospital System (RUHS) at Moreno Valley, CA, USA. A total of 11 (8 male and 3 female) human patients’ DFU lesion samples and nearby clean tissue samples were collected during surgery for TMA (Figure 1).

Patients with a history of smoking, myocardial ischemia, angina, macroalbuminuria, or any other serious illness were excluded. Informed consent from the patients was waived as the tissues were collected anonymously and informed consent taken for TMA surgery by the surgeon was suggested as sufficient by IRB. The tissue samples were collected in 10% buffered formalin for histology and immunostaining and in RNA later, following manufacturers’ instructions, for gene expression studies. The samples collected at RUHS were transported to Westen University of Health Sciences at Pomona, CA, USA at 4 °C and the samples in RNA later were stored at −80 °C, and in formalin were stored at room temperature.

Patient demographics: The mean age of the subjects was 55.81 years (range 45–72 years), and HbA1c levels were ranging between 5 to 12.8 with one patient with >14. The common aerobes identified in clean tissues were *S. viridans*, *S. aureus*, *S. pyogenes*, *Staphylococcus coagulase*, *Enterococcus species*, and *Enterococcus faecalis* in most of the patients except that 3 patient showed no growth. Clean tissues showed mixed anaerobes in one patient while the other patient showed no growth. The common aerobes in DFU tissues were *P. aeruginosa, S. Viridans, E. faecalis*, *S. pyogenes*, *S. coagulase*, *diphtheroid*, *and enterococcus species* in 10 patients while there was no growth in one patient. The positivity for these pathogens was different in each patient. DFU tissues showed mixed anaerobes. *Aspergillus niger*, *Candida albicans*, *Candida glabrata*, *Acremonium species* growth was detected in 5 patients while the remaining 6 patients did not show any growth. Patients were on anti-platelet, lipid-, blood pressure-, and glucose-lowering medication along with the medication for pain, neuropathy, and other disorders including psychological illness. Out of 11 patients, 3 had Type I (Insulin-Dependent) Diabetes Mellitus (IDDM) and 8 were suffering from type II diabetes mellitus (T2DM).

Tissue processing and histology: After keeping the tissues in formalin for 24 h, 4–5 mm tissue samples were processed using a tissue processor with multiple changes of formalin, ethanol, xylene, and paraffin wax. The tissues were embedded in paraffin blocks and 5 μm thin sections were sectioned using a Leica microtome. Tissue sections were kept on glass slides and incubated at 60 °C for one hour. For hematoxylin and eosin (H and E) staining, tissue sections were deparaffinized and hydrated with successive changes in xylene and ethanol (100%–95%–80%–75%) and stained with hematoxylin (45 s) and eosin (10 dips). This was followed by multiple changes in ethanol and the xylene followed by mounting with xylene-based mounting media Cytoseal. For trichrome staining, after deparaffinization and hydration, tissue sections were stained for trichrome staining using Masson trichrome staining kit (#HT15, Sigma Aldrich, St. Louis, MO, USA) and mounted using Cytoseal. All stained tissues were scanned using a DM6 Leica microscope with a scale of 100 μm. All scanned images were evaluated by two blind reviewers for inflammation, fibrosis, angiogenesis, necrosis, and vascular sclerosis in H and E-stained tissues and collagen and muscle staining on trichrome-stained sections.

Immunostaining: For immunohistochemistry (IHC), after deparaffinization and hydration, antigen retrieval was carried out using 1% citrate buffer and the slides were cooled down to room temperature. After this, the sections were washed with 1× phosphate-buffered saline (PBS) for 5 min and then the tissue sections were encircled using PepPen. Endogenous peroxidase blocking was done with 3% hydrogen peroxide (H1009; Sigma) for 15 min and the slides were rinsed in PBS for 5 min. This was followed by blocking with blocking solution (Vectastain Elite ABC kit; Vector Labs PK-6101; rabbit, PK-6105: goat, PK-6102: mouse) for 1 h at room temperature. Following this, the slides were incubated overnight at 4 °C with primary antibodies (Table 1) and then rinsed with 1× PBS three times for 5 min each. Then, the tissue sections were incubated at room temperature with biotinylated secondary antibody from Vectastain Elite ABC kit for 1 h. The tissue sections were rinsed 2 times with 1× PBS for five minutes each followed by incubation with Vectastain ABC horseradish peroxidase (HRP) for 30 min at RT. After rinsing with PBS, the tissue sections were incubated with AEC (3-amino-9-ethyl carbazole) Substrate Kit (Peroxidase (HRP), (SK-4200; Vector labs, Newark, CA, USA) for 2 to 5 min until the development of red color. After rinsing slides with water counterstain with hematoxylin was done and the sections were mounted using Cytoseal.

For immunofluorescence (IF), following de-paraffinization and rehydration, antigen retrieval was carried out using Epredia Lab Vision HIER Buffer L (TA135HBL, Fisher Scientific, Waltham, MA, USA). This was followed by blocking and incubation with primary antibodies (Table 1) overnight at 4 °C. The following day, tissue sections were washed and incubated with Alexa Fluor 488 and Alexa Fluor 596 secondary antibodies with a dilution of 1:1000 for 30 min. Vectashield Antifade Mounting Medium with DAPI (H-2000, Vector labs, Newark, CA, USA) was used for mounting the tissue sections. The stained sections were scanned using a Leica DM6 microscope with a scale of 100 μm. Stained intensity and area of staining in IHC and mean fluorescence intensity for IF was analyzed using ImageJ (NIH).

Total RNA isolation and Real-Time quantitative Polymerase Chain Reaction: Total RNA was isolated using the TRIZOL method (T9424, Millipore Sigma, Burlington, MA, USA) following standard procedure and the yield of total RNA was quantified using Nanodrop 2000 Spectrophotometer (Thermo Fisher, Waltham, MA, USA). Complementary DNA was prepared using iScript cDNA synthesis kit (BioRad) and qRT-PCR was carried out in triplicate using SYBR green with PCR cycling of 5 min at 95 °C for initial denaturation, 40 cycles of the 30 s each at 95 °C (denaturation), 30 s at 55–60 °C, and 30 s at 72 °C (extension) followed by melting curve analysis. The primers for genes of interest (Table 2) were purchased from Integrated DNA Technologies (Coralville, IA, USA). The relative gene expression was evaluated after normalization with 18S.

Statistical analysis: All data are presented as mean ± SD (standard deviation). Data were analyzed using GraphPad Prism 9. The comparison between two groups for the expression was performed using paired students’ t-test for statistical significance. A probability (*p*) value of <0.05 was accepted as statistically significant.

## 3. Results

**Figure 2:**
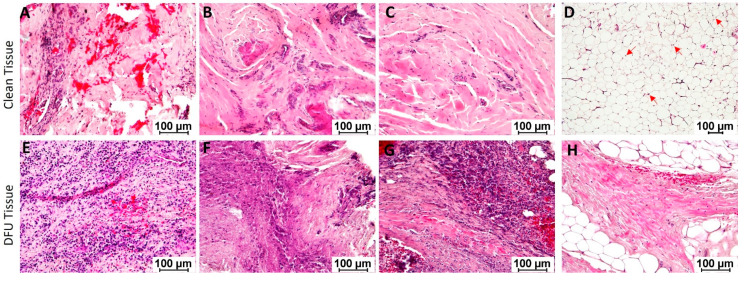
Hematoxylin and eosin staining in clean and diabetic foot ulcer (DFU) tissues. Clean tissues (**A**–**D**) and DFU tissues (**E**–**H**). The red arrows show the presence of a nucleus in adipocytes while most of the adipocytes in DFU tissues were without a nucleus. The images are representative images for both clean and DFU samples.

Hematoxylin and eosin staining: Hematoxylin and eosin (H and E) staining of the clean and DFU tissues showed mild to moderate focal inflammation in clean tissues while moderate to severe focal and generalized inflammation in DFU tissues (Figure 2, panels A–D vs. E–H). Neutrophils, macrophages, and lymphocytes were the main infiltrating immune cells. Additional findings were ischemic changes, fibrosis, and necrosis. The adipose tissues were viable with nuclei in clean samples and were not viable (without nuclei) in DFU samples.

**Figure 3:**
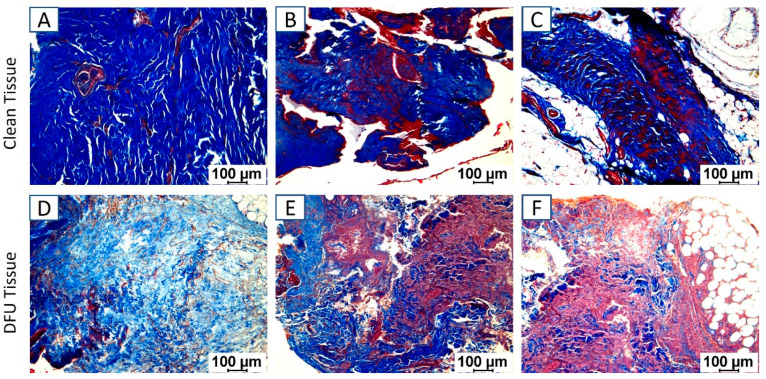
Masson’s trichrome staining in clean and diabetic foot ulcer (DFU) tissues. Clean tissues (**A**–**C**) and DFU tissues (**D**–**F**). The images are representative images for both clean and DFU samples.

Masson’s Trichrome staining: Masson’s trichrome staining of the clean and DFU samples revealed increased staining for collagen (blue) and muscle (red) in DFU samples (Figure 3D–F) compared to clean tissues (Figure 3A–C).

**Figure 4:**
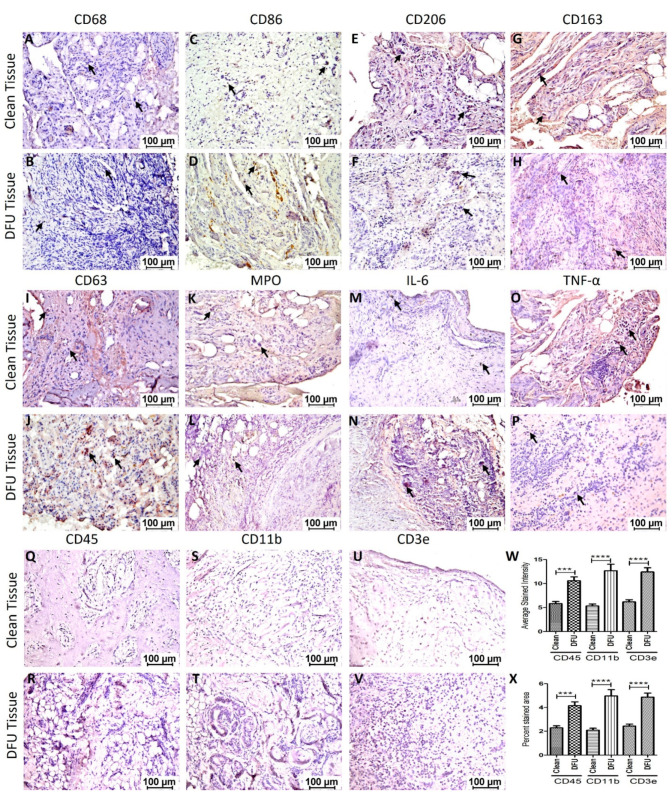
Immunostaining for mediators of inflammation and immune cell markers in clean and diabetic foot ulcer (DFU) samples. Macrophages (CD68, **A**,**B**), pro-inflammatory macrophages (M1 macrophage, CD86- **C**,**D**), anti-inflammatory macrophages (M2a macrophages, CD206- **E**,**F**); and M2b macrophages, CD163, **G**,**H**), activated neutrophils (CD63, **I**,**J**), neutrophils (myeloperoxidase-MPO- **K**,**L**), and pro-inflammatory cytokines IL-6 (**M**,**N**), TNF-α (**O**,**P**), CD45 (**Q**,**R**), CD11b (**S**,**T**), CD3e (**U**,**V**), and average stained intensity (**W**), and percent stained area (**X**). The images are representative images for both clean and DFU samples. The arrow shows stained cells in each panel. The data are presented as mean ± SD (*n* = 11 in each group). *** *p* < 0.001 and **** *p* < 0.0001.

**Figure 5:**
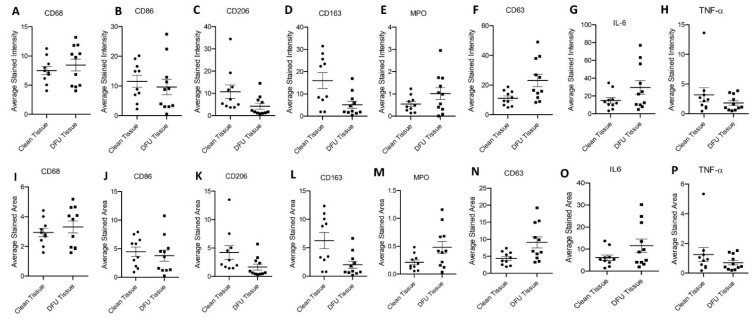
Mean stained intensity for CD68 (**A**), CD86 (**B**), CD206 (**C**), CD163 (**D**), MPO (**E**), CD63 (**F**), IL-6 (**G**), and TNF-(**H**) and average stained area for CD68 (**I**), CD86 (**J**), CD206 (**K**), CD163 (**L**), MPO (**M**), CD63 (**N**), IL-6 (**O**), and TNF-α (**P**) in clean and diabetic foot ulcer (DFU) tissues. The data are presented as mean ± SEM (*n* = 11 in each group).

Immunostaining: Immunostaining of the clean and DFU samples showed immunopositivity for macrophages (CD68), pro-inflammatory macrophages (M1 macrophage, CD86), anti-inflammatory macrophages (M2a macrophages, CD206, and M2b macrophages, CD163), neutrophils (myeloperoxidase-MPO), activated neutrophils (CD63), and pro-inflammatory cytokines IL-6 and TNF-α (Figure 4). Semiquantitative analysis of the average stained intensity and average stained area revealed increased average stained intensity for CD68, MPO, CD63, and IL-6 while decreased average stained intensity for CD86, CD206, CD163, and TNF-α in DFU tissues compared to clean tissues. The average stained area showed a similar trend of increased stained area for CD68, MPO, CD63, and IL-6 while decreased average stained area for CD86, CD206, CD163, and TNF-α in DFU tissues compared to clean tissues (Figure 5). Specifically, the stained intensity and area stained were much higher for CD206 and CD163 and much lower for MPO, CD63, and IL-6 in clean tissues compared to DFU tissues, though not significant. The expression of CD68, CD86, and TNF-α in both clean and DFU tissues followed the same tendency, but the expression was nearly the same. This may be due to a limited number of samples. However, surprisingly, the protein expression for lymphocytes (CD45, CD11b, and CD3e) was significantly higher in DFU tissues compared to clean tissues.

**Figure 6:**
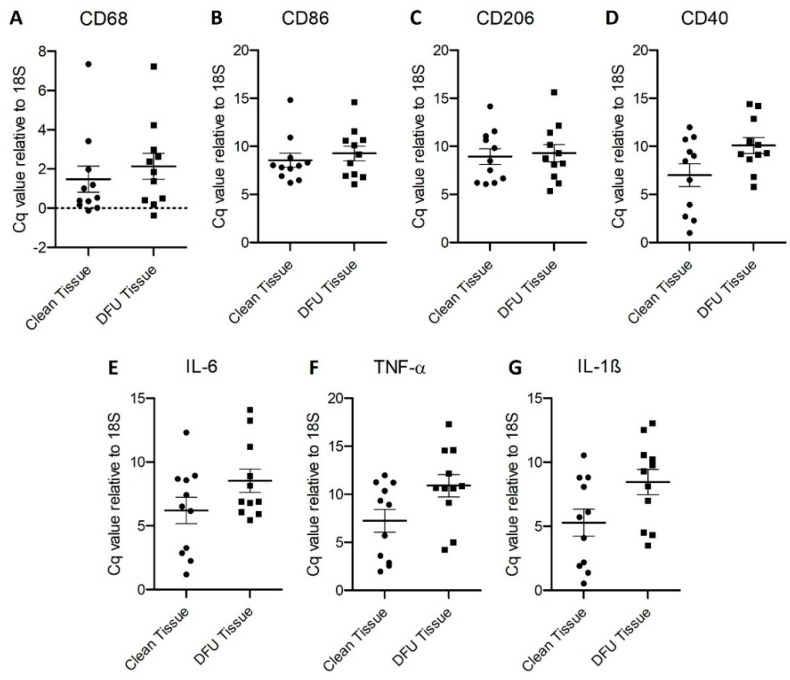
Real-time Polymerase Chain Reaction (RT-qPCR) for CD68 (**A**), CD86 (**B**), CD206 (**C**), CD40 (**D**), interleukin (IL)-6 (**E**), IL-1β (**G**), and tumor necrosis factor (TNF)-α (**F**). All data are presented as mean ± SEM (*n* = 11 in each group).

Real-Time Polymerase Chain Reaction: RT-PCR for the gene expression of CD68, CD86, CD206, CD40, IL-6, IL-1β, and TNF-α revealed increased relative gene expression (normalized to housekeeping gene 18S) in diabetic foot ulcer tissues compared to control tissues (Figure 6).

**Figure 7:**
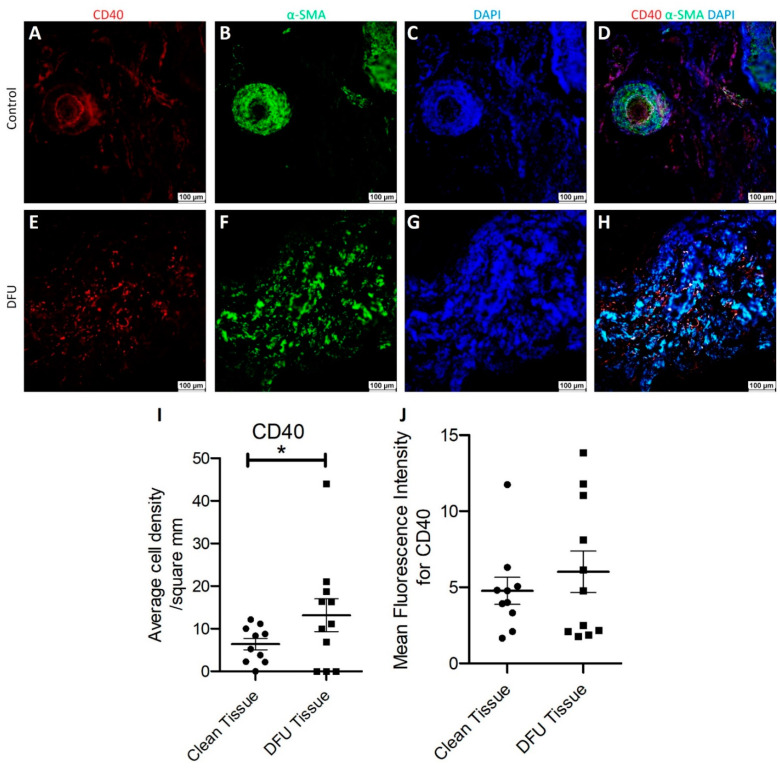
Dual immunofluorescence for alpha-smooth muscle actin (α-SMA) and CD40 in clean and diabetic foot ulcer (DFU) tissues. CD40 (**A**,**E**), α-SMA (**B**,**F**), DAPI (**C**,**G**), merged images (**D**,**H**), average cell density/mm^2^ (**I**), and mean fluorescence intensity for CD40 (**J**). All data are presented as mean ± SEM (*n* = 11 in each group). * *p* < 0.05.

Immunofluorescence: Dual-immunofluorescence of clean and diabetic ulcer tissues showed immunopositivity for both CD40 and α-SMA and some cells showed dual immunopositivity for both CD40 and α-SMA (Figure 7H). The dual immunopositivity was very minimal in clean tissue (Figure 7D), while it was significant in DFU tissue. These observations were supported by semiquantitative analysis of mean fluorescence intensity (MFI) analysis of CD40 stain showing increased MFI in DFU tissues compared to clean tissues. A significantly increased CD40+ cell density in DFU tissues compared to clean tissues (Figure 7I) further support our observation of the presence of CD40+ fibroblasts in the DFU tissues.

## 4. Discussion

The results of this study revealed the presence of chronic inflammation in both clean and DFU samples, but the inflammation was more severe in DFU samples compared to clean tissues. The presence of inflammation in the clean tissues is due to the site of tissue collection which was near to DFU area where acute inflammation might be mediating the healing response, but the presence of chronic inflammation suggests the presence of nearby nonhealing tissue. The presence of chronic inflammation in DFU tissues supports the notion that chronic inflammation contributes to the non-healing of DFUs [2,6,7]. Persistent inflammation mediated by infiltrating immune cells and secreted cytokines such as TNF-α induces activation of matrix metalloproteinases (MMPs) through mediators of inflammation and regulates collagen content [17], an important constituent of extracellular matrix and granulation tissue formation. Activation of MMPs in a chronic inflammatory state impairs the collagen content and due to an imbalance of collagen, I and collagen III impairs wound healing [18]. A decreased collagen and muscle expression in DFUs compared to clean tissues in association with chronic inflammation suggest that chronic inflammation-mediated increased proteases and elastases contribute to the nonhealing of DFUs [19].

Chronic inflammation is mediated by persistent infiltration of immune cells and secretion of proinflammatory cytokines [20] and the immunostaining and RT-PCR results showed immunopositivity for macrophages (CD68+, CD86+, CD206+, CD163+), neutrophils (MPO+ and CD63+), and pro-inflammatory cytokines (IL-6, IL-1β, and TNF-α) in DFU tissues compared to clean tissues. An increased positivity for CD68, MPO, and CD63 suggests the presence of macrophages (CD68), neutrophils (MPO and CD63), and activated platelets and granulocytes (CD63) [21] and is supported by the increased expression of pro-inflammatory cytokines IL-6, IL-1β, and TNF-α secreted from macrophages in DFU tissues. These findings support that chronic inflammation mediated by persistently infiltrated immune cells and secreted proinflammatory cytokines mediate impaired healing in DFUs [2,4,22,23]. An increased gene and protein expression of MPO and CD63 in DFU tissues support neutrophil-mediated chronic inflammation [24]. Inflammation in the DFU tissues may also be due to inflammation mediated by the adipose tissue with infiltrating immune cells [25,26] as revealed on hematoxylin and eosin staining.

The impaired healing response in diabetic tissues may also be due to imbalanced pro-and anti-inflammatory macrophages in the diabetic tissues. The results of this study showed an increased presence of pro-inflammatory macrophages in some patients while decreased in some patients compared to clean tissues. However, the expression of anti-inflammatory macrophages (CD206+ and CD163+) was lower in most of the DFU tissues compared to clean tissues. The variations in the expression of CD86 positivity may be due to a limited number of patients. Since a balanced number of pro-and anti-inflammatory macrophages during wound healing with a time continuum is important for a favorable inflammatory response and pro-inflammatory macrophages predominate during the early phase and anti-inflammatory macrophages during the late phase of the inflammatory response [27,28]. Fewer anti-inflammatory macrophages in DFU tissues suggest an inappropriate immune response in DFU tissues mediating nonhealing. Along with inflammatory cells, lymphocytes also play a role in nonhealing DFUs [29,30]. Impaired T-cell differentiation [31] and B-lymphocytes differentiation [32] are associated with chronic DFUs. A significantly increased expression and percent stained area of lymphocytes (CD45 and CD11b) and T-cells (CD3e) in nonhealing DFUs compared to clean tissues are per previous findings that lymphocytes play a critical role in DFU nonhealing.

Along with contributing to inflammation, immune cells interacting with fibroblasts can also regulate granulation tissue formation, ECM remodeling, immune response, and fibrosis [33]. This suggests that the presence of immune cells in the vicinity of fibroblasts affects fibroblast activity. This notion is supported by the fact that exposure of the fibroblasts to IL-1 and TNF-α leads to a change in phenotype switch to CD40+ subpopulation of fibroblasts (secretory fibroblasts) secreting increased amount of IL-6 and IL-8 [15,16]. This suggests that phenotypic change in fibroblasts may mediate impaired wound healing by mediating chronic inflammation through increased secretion of IL-6 and IL-8. Since, increased expression of IL-8 is associated with nonhealing DFUs [2,5], and fibroblasts acquiring CD40+ secretory phenotype secrete increased IL-6 and IL-8; it is imperative to correlate changing phenotype of fibroblasts in DFUs with nonhealing. This notion is also supported by the presence of five different populations of fibroblasts and changing the density of fibroblasts in nonhealing DFU [5,34]. The presence of CD40+ fibroblasts also positive for α-SMA (dual positive fibroblasts) supports the hypothesis of fibroblasts acquire secretory phenotype in DFUs and mediate chronic inflammation and impaired wound healing. This is because of a significantly increased number of CD40+ α-SMA+ fibroblasts in DFUs compared to clean tissues.

An important reason for the differential expression of various proteins in this study may be the ongoing treatment in these patients as well as the different infections and altered microbiota between clean and DFU tissues. As mentioned above, the patient showed different aerobes, anaerobes, and fungi in clean and DFU tissues. The main difference between patients was for aerobes in clean and DFU tissues. Altered gut microbiota is associated with the pathogenesis of type I and type II diabetes and associated complications [35,36]. Altered gut microbiota and gut–skin microbiota axis play a significant role in the inflammatory pathology of DFUs [37]. Further, studies have documented that even nonpathogenic bacteria may co-aggregate symbiotically and mediate chronic infection in nonhealing DFUs [38]. Moreover, the correlation of inflammation, cell membrane damage, cellular antigens, and protein metabolism with wound severity and microbiota function based on glycosylated hemoglobin (HbA1c) levels [39] suggest that altered microbiota and differential presence of microbes in each patient may pronounce different clinical outcome as well as the effect on various participating mediators. Sequencing analysis has revealed a high prevalence of anaerobes and Gram-negative bacteria in infected diabetic foot ulcers and the clinical outcome may be affected by specific genera, species, and bacterial functional genes [40]. These findings support the findings of the differential expression of various genes in this study. Furthermore, the recent research investigating the impact of commensal and pathogenic microbiota and the mechanisms of microbial–host communication in nonhealing DFUs to investigate novel therapeutic target [41] suggest the importance of altered microbiota in DFUs. Ongoing medications in these patients may also have contributed to the altered microbiota [42] due to the effect of medications on gut microbiota [43]. Another important interaction that should be considered while working on DFUs is the interaction between altered microbiota and its effect on the immune response. Activation, proliferation, and recruitment of immune cells including lymphocytes, macrophages, and neutrophils, and immune response may also be affected due to altered microbiota [44,45,46]. Another reason for the non-significance of the expression of various mediators may be the different types of tissues. The tissue samples were comprised of mainly muscles, adipose tissue, granulation tissue, and skin mainly in DFU tissues. Some tissues were a mixture of these while others were only adipose or muscle tissue.

These findings suggest that chronic inflammation and phenotypic change in the fibroblast population in DFUs may be of therapeutic significance. A phenotypic change of quiescent dermal fibroblasts to myofibroblasts is needed for proper wound healing because myofibroblasts secrete granulation tissue which is the foundation for angiogenesis [14,47]. Targeting fibroblast phenotypic switch is also important because dysregulated fibroblast function and plasticity and heterogeneity of fibroblasts play a critical role in DFU wound healing [47,48,49]. Targeting the fibroblasts in a chronic inflammatory environment is also important because TNF-α affects α-SMA expression in dermal fibroblasts [50]. Finally, targeting phenotypic change in fibroblast in DFU wound healing is supported by the fact that fibroblast phenotypic switch plays a critical role in wound healing after myocardial infarction having similar pathological conditions of wound healing as in DFU [51,52,53]. Further, the effect of altered microbiota on fibroblast phenotype and inflammation should be considered.

## 5. Conclusions

**Figure 8:**
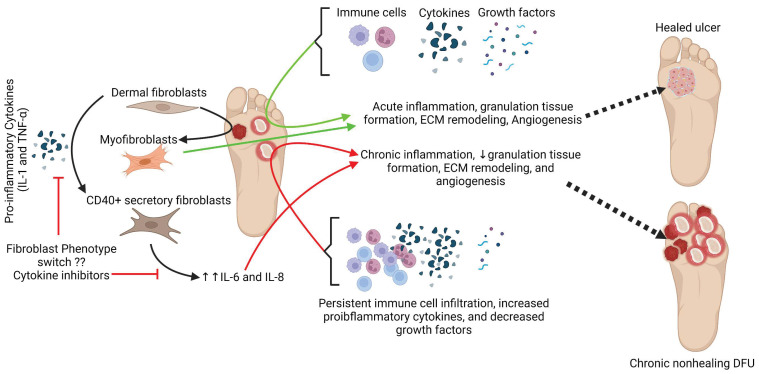
Schematics showing the pathogenesis of chronic inflammation-mediated impaired wound healing and phenotypic change of fibroblasts in an inflammatory environment. Interleukin (IL), tumor necrosis factor (TNF), and extracellular matrix (ECM).

Overall, the findings of this study elucidated the presence of CD40+ fibroblasts, for the first time, in DFUs in association with chronic inflammation. Since the exposure of fibroblasts with IL-1 and TNF- α mediate the phenotypic change in fibroblasts which mediate increased secretion of other inflammatory cytokines, targeting the fibroblast phenotypic switch will be of therapeutic significance (Figure 8). It is integrative to think that increased CD40+ fibroblasts might be at the cost of myofibroblasts leading to decreased granulation tissue formation and angiogenesis.

## 6. Limitations of the Study

This study provides significant results on the presence of CD40+ fibroblast in DFUs which may contribute to chronic inflammation and nonhealing. A limited number of the tissues, necrotic tissues, and different types of collected tissues (muscle, adipose, fibro adipose, and skin), infections, and other patient-related factors may have confounded the results. Despite these limitations, this study has shown for the first time the presence of CD40+ fibroblast and the possibility of targeting fibroblast phenotype switch to promote wound healing.

## Figures and Tables

**Table 1:** Primary antibodies and the dilution factor used for immunostaining and immunofluorescence in this study.

Antibody	Catalog #	Dilution
CD68	ab955	1:200
CD86	ab269587	1:100
CD206	ab64693	1:200
CD163	ab87099	1:200
MPO	sc-365436	1:50
CD63	ab134045	1:100
CD45	ab10558	1:50
CD11b	ab8878	1:200
CD3e	ab5690	1:100
IL-6	ab6672	1:100
TNF-α	ab6671	1:200
CD-40	ab224639	1:200
A-SMA	ab5694	1:50

MPO—myeloperoxidase, IL—interleukin, TNFα—tumor necrosis factor-alpha, α-SMA—alpha-smooth muscle actin. #—indicate the “number”.

**Table 2:** Forward and reverse nucleotide sequences used for the gene of interest in a quantitative real-time polymerase chain reaction (qRT-PCR).

Gene Name	Forward	Reverse
CD68	5’-ACGGCTCATGCCTGTAATC-3’	5’-GCCACACCTGGCTAATTGTA-3’
CD86	5’-CTAGGGTACAGGCAACAATGAG-3’	5’-TTAGCAACAGCCCAGATAGAAG-3’
CD206	5′-TTGGACGGATAGATGGAGGG-3′	5′-CCAGGCAGTTGAGGAGGTTC-3′
CD40	5′-GTCGGCTTCTTCTCCAATGT-3′	5′-TGATAAAGACCAGCACCAAGAG-3′
IL-6	5′-ATAGGACTGGAGATGTCTGAGG-3′	5′-GCTTGTGGAGAAGGAGTTCATAG-3′
TNF-α	5′-AGGCGCTCCCCAAGAAGACA-3′	5′-TCCTTGGCAAAACTGCACCT-3′
IL-1β	5′-ATGGACAAGCTGAGGAAGATG-3′	5′-CCCATGTGTCGAAGAAGATAGG-3′

Cluster of differentiation (CD), interleukin (IL), and tumor necrosis factor (TNF)-α.

## Data Availability

All data related to this manuscript have been included in this manuscript. The raw data can be obtained on request from the corresponding author.
